# Analyses and interpretation of whole-genome gene expression from formalin-fixed paraffin-embedded tissue: an illustration with breast cancer tissues

**DOI:** 10.1186/1471-2164-11-622

**Published:** 2010-11-08

**Authors:** Muhammad G Kibriya, Farzana Jasmine, Shantanu Roy, Rachelle M Paul-Brutus, Maria Argos, Habibul Ahsan

**Affiliations:** 1 Department of Health Studies, The University of Chicago, 5841 S. Maryland Avenue, MC 2007, Chicago, IL 60637, USA; 2 Department of Human Genetics, The University of Chicago, Chicago, IL 60637, USA; 3 Department of Medicine, The University of Chicago, Chicago, IL 60637, USA; 4 Comprehensive Cancer Center, The University of Chicago, Chicago, IL 60637, USA

## Abstract

**Background:**

We evaluated (a) the feasibility of whole genome cDNA-mediated Annealing, Selection, extension and Ligation (DASL) assay on formalin-fixed paraffin-embedded (FFPE) tissue and (b) whether similar conclusions can be drawn by examining FFPE samples as proxies for fresh frozen (FF) tissues. We used a whole genome DASL assay (addressing 18,391 genes) on a total of 72 samples from paired breast tumor and surrounding healthy tissues from both FF and FFPE samples.

**Results:**

Gene detection was very good with comparable success between the FFPE and FF samples. Reproducibility was also high (r^2^ = 0.98); however, concordance between the two types of samples was low. Only one-third of the differentially expressed genes in tumor tissues (compared to corresponding normal) from FF samples could be detected in FFPE samples and conversely only one-fourth of the differentially expressed genes from FFPE samples could be detected in FF samples. GO-enrichment analysis, gene set enrichment analysis (GSEA) and GO-ANOVA analyses also suggested small overlap between the lead functional groups that were differentially expressed in tumor detectable by examining FFPE and FF samples. In other words, FFPE samples may not be ideal for picking individual target gene(s), but may be used to identify some of the lead functional group(s) of genes that are differentially expressed in tumor. The differentially expressed genes in breast cancer found in our study were biologically meaningful. The "cell cycle" & "cell division" related genes were up-regulated and genes related to "regulation of epithelial cell proliferation" were down-regulated.

**Conclusions:**

Gene expression experiments using the DASL assay can efficiently handle fragmentation issues in the FFPE tissues. However, formalin fixation seems to change RNA and consequently significantly alters gene expression in a number of genes which may not be uniform between tumor and normal tissues. Therefore, considerable caution needs to be taken when interpreting gene expression data from FFPE tissues, especially in relation to specific genes.

## Background

High-throughput microarray technology is a powerful tool for genome-wide genotyping and gene expression analysis. Microarray-based gene expression assessment is a very useful method for prediction of diseases, tumor classification and drug responses. Although good quality RNA can be extracted from fresh frozen (FF) tissues, tissues preserved in RNAlater reagent and primary cell culture, the limited availability of these sources is a problem with regards to the utility of gene expression measurements. As Formalin-Fixed Paraffin-Embedded (FFPE) sample collection and storage are routine practices in pathological laboratories worldwide, there is great interest in the use of RNA extracted from those archived samples.

Integrity of nucleic acid is a very important issue for microarray analysis. It is well known that the FFPE tissue RNA is often degraded and at the same time, it is chemically modified [1,2]. Previous studies using different microarray platforms showed that only approximately 3% or less of the RNA isolated from the FFPE tissue are useful for cDNA synthesis, which is an important step for gene expression analysis on a microarray platform [3]. Illumina Inc. introduced a gene expression profiling method, DASL (cDNA-mediated Annealing, Selection, extension and Ligation) specially designed for analysis of fragmented RNA samples [4-6]. In the present study, we used the Whole Genome DASL Assay on paired breast tumor and surrounding healthy tissue from both FFPE tissue and FF tissue to examine: (a) the feasibility of genome-wide gene expression analyses using DASL on FFPE tissue and (b) whether similar conclusions can be drawn by examining widely available FFPE tissue as a proxies for FF tissues.

## Methods

### Tissue Samples

The study was approved by the Institutional Review Board of The University of Chicago. We obtained four different sets of tissue samples (paired tumor and adjacent normal breast tissue from fresh frozen as well as corresponding FFPE blocks) from the same patients through our Human Tissue Resource Center (HTRC) at The University of Chicago http://pathcore.bsd.uchicago.edu/BSB/BSB_overview.shtml. HTRC collected these breast tissues from mastectomy/lumpectomy specimens that were not needed for pathological diagnosis by the Department of Pathology at The University of Chicago Medical Center. There were a total of 21 such patients for whom all 4 types of tissues were available. For the present study, tissue samples from the first 18 patients were used. These tissue samples were archived for a 3-6 year period.

### RNA extraction and quality control

For the extraction of total RNA from both FFPE and FF breast tissue samples, we used the High Pure RNA Paraffin Kit (Roche Applied Science, cat# 03 270 289 001). Xylene was used for deparaffinization of the FFPE samples. Proteinase K digestion was carried out by overnight incubation at 55°C. DNAse treatment was carried out for all of the samples and samples were eluted with 40 μl of the elution buffer provided with the extraction kit. For quantification, each sample was (a) tested in a NanoDrop 1000 for concentration and 260/280 ratio and also (b) run in an Agilent Bioanalyzer using the Eukaryotic total RNA Nano Series II kit. Prequalification of all the RNA samples derive from FFPE and FF tissue was done by qPCR using SYBR Green detection. cDNA was synthesized as recommended by Illumina, and then a 90 bp amplicon was amplified from the highly expressed *RPL13a *ribosomal protein gene (GenBank Accession # NM_012423.2). The amplification reaction was carried out for all FFPE and FF samples in duplicates in ABI Prism 7900 HT Sequence Detection Systems (Applied Biosystems). The ΔCt was calculated as Ct (test sample) - Ct (control sample). As per Illumina protocol for DASL, a ΔCt of up to 12 was acceptable as prequalification for the assay. Only 4 out of 72 breast tissue samples had ΔCt just above 12 and none > 12.5. Only 1 of these 4 samples showed a poor performance on the assay (see result section).

### Whole genome DASL assay

We used 300 ng of total RNA (5 μL at 60 ng/μL concentration) as starting material for each sample run on the microarray. Each chip accommodates 8 samples. Four types of sample for each patient were processed in one chip. The four types of sample were FF tumor tissue (T_FF), FF adjacent normal tissue (N_FF), FFPE tumor tissue (T_FFPE) and (4) FFPE adjacent normal tissue (N_FFPE). Thus, a single chip contained 8 samples from 2 patients and three such chips (24 samples) were processed in one batch. We also ran four technical replicates of the T_FFPE RNA samples to analyze the reproducibility of the DASL assay. According to the manufacturer's protocol, RNA was first converted to cDNA through reverse transcription with biotinylated primers. The cDNA was then annealed to the assay oligonucleotide and bound to streptavidin conjugated paramagnetic beads. After the oligohybridization, non-hybridized and miss-hybridized sequences were washed away and the hybridized sequences were extended and ligated to start PCR amplification with fluorescent tagged primers. The fluorescently labeled amplified PCR products were hybridized overnight onto the bead chip to be scanned on Bead Array reader.

### Statistical analyses and power

#### Statistical analysis

To compare the continuous variables (e.g. number of detected genes/samples or average signal intensity across different groups), we used one-way analysis of variance (ANOVA). For the gene expression data, we used quantile normalization in BeadStudio software before exporting the data for PARTEK Genomic Suite [7] for further statistical analyses. Principle components analysis (PCA) and sample histograms were checked as a part of quality control analyses of the data. Mixed-model multi-way ANOVA (which allows more than one ANOVA factor to be entered in each model) was used to compare the individual gene expression data across different groups. In general, "disease status" (tumor/adjacent normal) and "storage method" (FFPE/FF) were used as categorical variables with fixed effect since the levels tumor/normal or FFPE/FF represent all conditions of interest; whereas "case ID#" (as proxy of inter-person variation) and "batch#" were treated as categorical variable with random effect, since the person or batch are only a random sample of all the levels of that factor. Method of moments estimation was used to obtain estimates of variance components for mixed models [8]. As per study design we processed all four types of samples from one individual in a single chip (i.e., in same batch), therefore batch# and case ID# were linked, and thus they were not entered in the same model. In combined analysis when we included FFPE and FF samples together, the log_2_-transformed signal intensity was used as the response variable, and "tissue type " (or "disease" i.e. tumor or normal), "sample storage" (i.e. FFPE or FF) and case ID# were entered as ANOVA factors. In order to examine interaction between "tissue type" and "sample storage", we also entered the cross-product term "tissue type" × "sample storage" in the 3-way ANOVA model. When we separately analyzed the FF and FFPE samples to identify differentially expressed genes in breast tumor compared to adjacent normal tissue, disease status or "tissue type", "batch#" and "block archival age" were entered as ANOVA factors in the 3-way ANOVA model. One example of a model [9] that uses method of moments for combined analysis is as follows:

$${\text{Y}}_{\text{ijkl}}=\mu +{\text{Tissue}}_{\text{i}}+{\text{Storage}}_{\text{j}}+{\text{Case ID}}_{\text{k}}+{\text{Tissue*Storage}}_{\text{ij}}+\varepsilon_{\text{ijkl}}$$

Where Y_ijkl _represents the gene expression intensity of gene "Y" in l-th sample with i-th tissue with j-th storage for the k-th case ID; μ is the common effect for the whole experiment; and ε_ijkl _represents the random error, which is assumed to be normally and independently distributed with mean 0 and standard deviation δ for all measurements. Case ID is a random effect in this model.

Similarly, when we analyzed the data separately for FF and FFPE samples to detect individual genes differentially expressed in tumor, we used the following 3-way ANOVA model by using method of moments:

$${\text{Y}}_{\text{ijkl}}=\mu +{\text{Tissue}}_{\text{i}}+{\text{Batch no}}_{\text{j}}+{\text{Block\_Age}}_{\text{k}}+\varepsilon_{\text{ijkl}}$$

Where Y_ijkl _represents the gene expression intensity of gene "Y" in l-th sample with i-th tissue for the j-th Batch no with k-th Block_Age. As in the model for combined analyses above, μ is the common effect for the whole experiment and ε_ijkl _represents the random error, which is assumed to be normally and independently distributed with mean 0 and standard deviation δ for all measurements. Batch no is a random effect in this model.

In GO Enrichment analysis, we tested if the genes found to be differentially expressed fall into a Gene Ontology category more often than expected by chance. We used Chi-square test to compare "number of significant genes from a given category/total number of significant genes" vs. "number of genes on chip in that category/total number of genes on the microarray chip". Negative log of the p-value for this test was used as the enrichment score. Therefore, a GO group with a high enrichment score represents the lead functional group. The enrichment scores were analyzed in a hierarchical visualization and in tabular form.

In addition to looking at differential expression at individual gene level, we also examined the differential expression of gene sets using the Gene Set Enrichment Analysis (GSEA) [10]. Given an a priori defined set of genes S (sharing the same GO category), the goal of GSEA was to determine whether the members of S were randomly distributed throughout the ranked list or primarily found at the top or bottom. Considering the fact that GSEA can look at single variable (unadjusted expression), we also used GO-ANOVA that offers adjustments for other factors like "person-to-person" variation, "tissue type" variation etc.

GO-ANOVA is a mixed model ANOVA to test the expression of a set of genes (sharing the same GO category) instead of an individual gene in different groups [7]. The analysis is performed at the gene level, but the result is expressed at the level of the GO-category by averaging the member genes' results. The equation for the model was:

Model:Y=μ+T+P+G+S(T*P)+ε

Where Y represents expression of a GO-category, μ is the common effect or average expression of the GO-category, T is tissue-to-tissue (tumor/healthy) effect, P is patient-to-patient effect, G is gene-to-gene effect (differential expression of genes within the GO-category independent of tissue types), S(T*P) is sample-to-sample effect (this is a random effect, and nested in tissue and patient) and ε represents the random error.

Cross-validation: For the one-level cross validation, the data was first divided into 10 random partitions. At each iteration, 10% samples were held out for testing while the remaining 90% samples were used to fit the parameters of the model. We also used a 10 × 10 two-level nested cross-validation [11]. In the outer cross-validation, with 10% of samples held out as test cases, the remaining 90% were used in a 10-fold cross-validation to determine the optimal predictor variables and other classifier parameters. The model that performed the best on the inner cross-validation was applied to the held-out test samples in the outer cross-validation. Thus an inner cross-validation was performed in order to select predictor variables and optimal model parameters, and an outer cross-validation was used to produce overall accuracy estimates for the classifier. In the first step, we considered all the differentially expressed genes for inclusion in the model and then in the next step(s), for selecting the top 50 or 100 genes, expression of which could be used to differentiate the FFPE samples from the FF samples, we used 3-way ANOVA ["storage" (FFPE or FF) adjusted for "tissue type" (tumor or normal) and "person-to-person" variation]. We tested three classification methods - (a) K-Nearest Neighbor (KNN) with Euclidean distance measure and 1-neighbor, (b) nearest centroid with equal prior probability and (c) linear discriminent analysis with equal prior probability.

#### Statistical power

In genome-wide gene expression experiments requiring multiple testing, it is more powerful and more reasonable to control false discovery rate (FDR) or positive FDR (pFDR) [12-14] instead of type I error. We followed that strategy for sample size calculation. When controlling FDR, the traditional approach of estimating sample size by controlling type I error is no longer applicable [15]. In their paper, Liu P and Hwang JTG have compared calculations of sample size using four different approaches - all of which had good agreement and show that, for standardized effect size Δ/σ = 1 [e.g., for fold change of 1.4 with σ = 0.5; Δ/σ = (log_2 _1.4)/0.5) = 1], if identification of 95% of the truly altered genes are desired (our set target), then the estimated sample size for each group would be between 33 and 34. For standardized effect size Δ/σ = 2 [in other words, for fold change of 2 with σ = 0.5; as Δ/σ = (log_2 _2)/0.5) = 2], if identification of 95% of the truly altered genes is desired (our set target), then the estimated sample size for each group would be 11. Therefore, our study was sufficiently powered to detect a 1.4-fold change difference in the combined analysis and a 2-fold change in the subgroup analysis where FF and FFPE samples were analyzed separately.

## Results

Performance of the assay, evaluated by the number of detected genes per sample, was impressive. Among the 18,391 genes on the DASL assay, on average, about 11290 genes (61.4%) were detected in each sample at p < 0.05 level. A gene was said to be detected at p < 0.05 level if the mean signal intensity from multiple probes for that gene was significantly higher (at the level of p < 0.05) than the negative control on the same chip. Table 1 shows that the number of genes detected per sample at p < 0.05 level per sample was comparable in all the four tissue types (N_FF, T_FF, N_FFPE and T_FFPE), suggesting uniform performance of DASL across FF and FFPE samples stratified by tissue type. The average signal intensity for all the genes and for the housekeeping genes are also shown in Table 1.

**Table 1:** Sample characteristics and assay performance

		*N*	*Mean*	*95% Confidence Interval for Mean*		*ANOVA*
				**Lower Bound**	**Upper Bound**	**Sig**.

Detected Genes (0.05)	N_FF	18	11274.94	10750.46	11799.43	0.788559493
	T_FF	18	11283.50	10278.99	12288.01	
	N_FFPE	18	11063.11	10498.01	11628.21	
	T_FFPE	18	11542.39	10951.83	12132.94	
	Total	72	11290.99	10965.42	11616.55	
Signal Average	N_FF	18	1980.32	1972.98	1987.65	1.37991E-10
	T_FF	18	1951.04	1939.61	1962.46	
	N_FFPE	18	1932.33	1922.32	1942.33	
	T_FFPE	18	1929.77	1918.58	1940.97	
	Total	72	1948.36	1941.68	1955.05	
HOUSEKEEPING	N_FF	18	6879.53	5605.81	8153.24	0.168579448
	T_FF	18	7897.44	6729.16	9065.73	
	N_FFPE	18	6620.02	5638.74	7601.30	
	T_FFPE	18	8180.73	6767.25	9594.20	
	Total	72	7394.43	6809.35	7979.51	
Block_Age	N_FF	18	3.83	3.44	4.22	1
	T_FF	18	3.83	3.44	4.22	
	N_FFPE	18	3.83	3.44	4.22	
	T_FFPE	18	3.83	3.44	4.22	
	Total	72	3.83	3.65	4.01	
Difference of CT	N_FF	18	7.45	6.69	8.21	0.011648246
	T_FF	18	8.36	7.27	9.45	
	N_FFPE	18	9.53	8.47	10.59	
	T_FFPE	18	7.87	7.00	8.74	
	Total	72	8.30	7.83	8.78	

### Reproducibility

**Figure 1:**
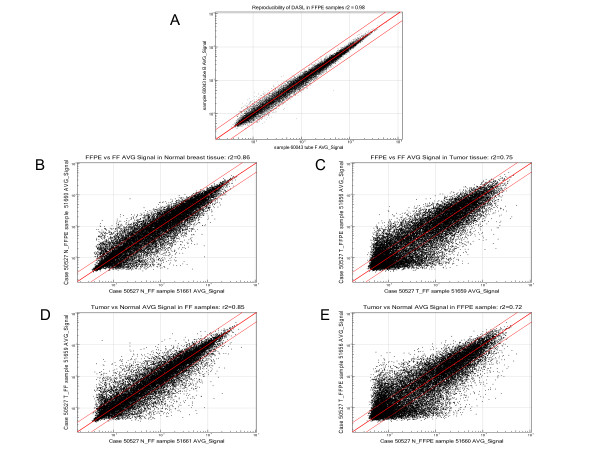
**Scatter plot of the log_2_-transformed signal intensities**. Scatter plot of the log_2_-transformed signal intensities of representative samples are shown in 1A - 1E. The central straight line represents the regression line and the two lines on the either sides mark the boundaries for 2-fold changes. Pearson correlation coefficient squared (r^2^) for each scatter plot is shown on the top. Figure-1A shows the signal intensities of two technical replicates of RNA samples. Figure-1B & 1C show correlations between corresponding FF (x-axis) & FFPE samples (y-axis) in normal breast tissue and tumor tissue respectively. Figure 1D & 1E show correlations between corresponding tumor (y-axis) and normal (x-axis) tissue from same patient using FF and FFPE samples respectively.

We ran 4 samples in duplicate (technical replicates) to check the reproducibility. A representative figure showing the correlation between log_2 _signal intensities is presented in Figure 1A which shows r^2^ = 0.98, suggesting acceptable reproducibility. The central line represents the regression line and the two lines on either side of the central line represent the boundary for 2-fold change.

Correlations of log_2 _signal intensity between a pair of RNA samples extracted from N_FF and N_FFPE tissue (r^2 ^= 0.86) and between T_FF & T_FFPE tissue (r^2^ = 0.75) from same subject (case ID#50527) are shown in Figure 1B and Figure 1C respectively. Both the graphs show that in normal as well as in tumor tissue, a number of genes were found to be more than 2-fold up- or down-regulated in FFPE samples compared to corresponding FF samples. Similarly, correlations between signal intensity from paired RNA samples extracted from N_FF and corresponding T_FF tissue (r^2^ = 0.85) and paired RNA samples extracted from N_FFPE and T_FFPE tissue (r^2^ = 0.72) from the same subject (case ID#50527) are shown in Figure 1D and Figure 1E respectively. As expected, in both the FF and FFPE samples, there were a number of genes that were more than 2-fold up- or down-regulated in the tumor tissue compared to the corresponding normal tissue.

### Sources of variation in the gene expression

**Figure 2:**
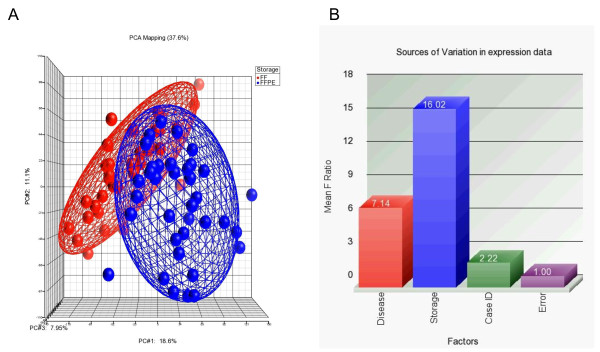
**Principle components analysis (PCA) and sources of variation**. Principle components analysis (PCA) of 71 samples displaying spatial separation (expression level clustering) of RNA extracted from FF samples (shown in red on left) and FFPE samples (shown in blue on right); The three principle components (PC#1, #2 & #3 and respective contributable variations are presented on x-, y- and z-axis along). A Statistical significance of the different "sources of variation" in the total gene expression estimated by 3-way ANOVA models. F ratio for each factor (source) represents the F-statistics for that factor/F-statistics for error (noise). The mean of the F Ratio for all the genes for a particular factor are shown on Y-axis and the different factors (sources of variation) are shown in x-axis.

As part of the quality control of the data, we utilized principle component analysis (PCA) which indicated only one sample as an outlier. This sample had only 4606 genes detected at p < 0.05 level and also had a ΔCt = 12.36. We excluded this sample from further analysis. PCA on the remaining 71 breast tissue samples is shown in Figure 2A, where the samples are color-coded by of sample storage type. The figure suggests a clustering effect of sample storage type. When we color-coded the samples by tissue type (normal or tumor, i.e. "disease"), we also saw a clustering effect of tissue type, but less prominent than for the type of sample storage.

In the next step, to further investigate the source of variation in the expression, we used multivariate ANOVA. We did not use any filter for selecting the genes to be included in the analysis. In other words, ANOVA was performed on log_2_-transformed intensity value for all the 18,391 genes irrespective of their level of detection. The average F-ratio (F-statistics for the test variable/F-statistics for error) for all the genes was considered as representative of significance of signal-to-noise ratio. Figure 2B shows the significance of different sources of variation in the entire data in a model where tissue type (tumor/normal), sample storage (FFPE/FF) and person-to-person variation (case ID#) were entered as explanatory variables at a time for gene expression. The figure shows that "sample storage" (F ratio 16.02) was the most significant source of variation, followed by the "disease status" (F-ratio 7.14) and person-to-person variation (F-ratio 2.22).

### Differential gene expression in FFPE samples compared to FF

**Figure 3:**
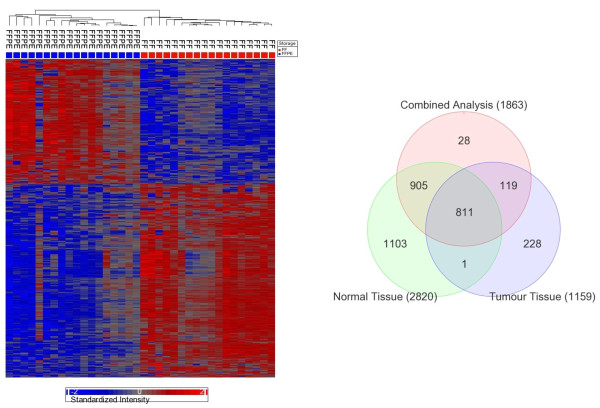
**Unsupervised hierarchical clustering and lists of differentially expressed genes in FFPE**. **A. **Heat map of hierarchical clustering. Normal breast tissue samples in FFPE (color coded and labeled at the top) and corresponding FF samples are shown in columns. Log_2 _transformed signal intensities of 2820 differentially expressed genes are shown in color code (bar at bottom) in rows. **B. **Venn diagram showing overlap between the three lists of the differentially expressed genes (at FDR 0.05 level and at least 2-fold) in FFPE compared to FF samples from three different analyses - normal breast tissue only (bottom left showing 2820 genes), corresponding breast tumor tissue only (bottom right showing 1159 genes) and combined analysis of normal & tumor tissue (top center showing 1863 genes).

In the total set of 71 breast samples, we looked at the genome-wide (n = 18,391) differential gene expression in FFPE samples compared to FF samples after adjustment for tissue type (tumor/normal), and inter-person variation. There were a total of 9186 differentially expressed genes (49.94% of 18391) at FDR 0.05. Concerning fold change, a total of 1864 genes had > 2-fold change in either direction (1098 up- and 766 down-regulated). Combining these two criteria, there were a total of 1863 genes (10.12% of total 18,391 genes) that were differentially expressed by at least 2-fold at FDR 0.05 level (766 up- and 1097-down-regulated in FFPE). For the statistically most significantly (p = 2.17 × 10^-28^) differentially expressed gene, GIGYF1, 83.6% of the variation was due to "storage". Taking all of these 1,863 differentially expressed genes in FFPE samples into account, we found that overall 30.32% of the variation in individual gene expression was contributed by "storage" (FFPE or FF), 4.94% by tissue type (tumor or normal), 25.43% by "person-to-person" variation and the rest 39.31% of the variation could not be explained by the ANOVA model. It may be noted that among these 1,863 genes, a total of 138 genes (7.4%) were also differentially expressed (at least two-fold change) in tumor tissue compared to healthy tissue at FDR 0.05 in our data set. In the Breast Cancer Gene Database (BCGD) [16], there was a total of 62 genes of which 51 were studied in our gene-chip. We also looked if the expression of any of those 51 breast cancer related genes in BCGD list was also found to be affected by FFPE in our setting. In fact five of them (APC, CDKN2A, IGF1R, TGFA and TSG101) were in our list. We also separately tested the normal and tumor tissue data to see the effect of FFPE preservation. In the analysis of normal tissue only, a total of 2820 genes in FFPE samples were differentially expressed (1712 down- and 1108 up-regulated) at least by 2-fold at FDR 0.05 level, compared to corresponding FF samples. Cluster analysis based on these genes could effectively separate the FFPE samples from the FF samples (see Figure 3A). In the analysis of tumor tissue only, a total of 1159 genes in FFPE samples were differentially expressed (555 down- and 604 up-regulated) at least by 2-fold at FDR 0.05 level, compared to corresponding FF samples. Cluster analysis based on these genes also could effectively separate the tumor FFPE samples from the tumor FF samples (figure not shown). The Venn diagram in Figure 3B shows the overlap among these gene lists obtained from (a) combined analysis, (b) normal tissue alone and (c) tumor tissue alone to look for differentially expressed genes in FFPE tissue compared to corresponding FF tissue.

#### Cross-validation

A 10 × 10 two-level nested cross-validation analysis using all the 1,863 differentially expressed genes in FFPE in present study suggested that the expression of these genes could differentiate FFPE samples from FF samples with overall accuracy of 95.7%. The same 10 × 10 two-level nested cross-validation analysis suggested that the expression of the top 100 genes could be used in a model that could differentiate FFPE samples from FF samples with overall accuracy of 100%. We also tested the top 50 genes, and again the overall accuracy was 100%. This further supported our ANOVA finding that a number of genes are differentially expressed in FFPE samples compared to their corresponding FF sample.

### Differential expression profile at "gene set" level in FFPE samples compared to corresponding FF

**Figure 4:**
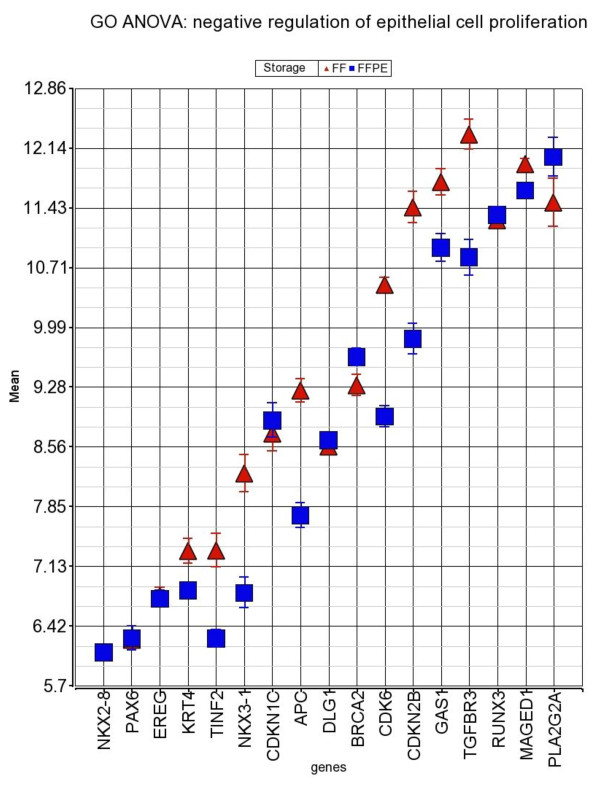
**Result from "gene set" analysis - GO-ANOVA**. GO-ANOVA result for Differential gene expression of the gene set "negative regulation of epithelial cell proliferation" in FFPE. The members of this category are shown in x-axis and the corresponding log_2_-transformed expression is presented on y-axis; error bar represents SE.

In addition to individual gene level analysis, we also performed GSEA to find out "gene sets" that are up- or down-regulated in FFPE. The top five "gene sets" (each representing a GO-category) that were down-regulated in FFPE include: "nucleotide excision repair, DNA gap filling" (normalized enrichment score- NES 2.11), "negative regulation of epithelial cell proliferation" (NES 1.98), "chaperone binding", "DNA polymerase activity" and "DNA-directed DNA polymerase activity". The up-regulated GO-category includes: "positive regulation of gene-specific transcription" (NES -1.95), "regulation of Rho protein signal transduction", "Rho guanyl-nucleotide exchange factor activity", "dopamine receptor signaling pathway", and "thymic T cell selection". GO-ANOVA also confirmed these findings. Figure 4 shows one of the examples: the genes in GO-category "**negative regulation of epithelial cell proliferation**" (p = 6.65 × 10^-14^) were overall down-regulated in FFPE.

Considering the effect of FFPE on the gene expression, we then looked at the differential gene expression in tumor tissue compared to adjacent normal tissue in two ways - (a) looking at the combined data (FF and FFPE) with adjustment for "sample storage" and "person to person variation" and (b) looking at the FF and FFPE data separately with paired comparisons to detect differential expression in tumor and to see if similar conclusion(s) could be made.

### Differential gene expression in breast tumor tissue compared to corresponding adjacent normal breast tissue

Among the 18 individuals included in the analyses of breast tissues, one had fibroadenoma, 3 had no abnormality detected and in one the histopathology was unknown. Therefore, to get a clearer picture of genes differentially expressed in breast cancer, we included 52 samples from 13 patients with known histopathological diagnosis of breast cancer (4 samples from each patient). Of these 52 samples, one FF tumor sample was excluded due to poor performance on the chip (an outlier on PCA, as mentioned above). The analysis was restricted to the 51 arrays. In addition to PCA, we also used unsupervised hierchical clustering based on all 18,391 genes which showed clustering by sample storage (FF/FFPE) as well as by histopathology [figure not shown].

In this total set of 51 samples, we looked at the genome-wide (n = 18391) differential gene expression in breast tumor tissue compared to adjacent normal tissue after adjustment for "sample storage" (FF/FFPE), and "person to person variation" (case ID#). There were a total of 1319 genes (7.17% of total 18,391 genes; 604 were up-regulated and the rest 715 were down regulated in tumor) that were differentially expressed at least by 2-fold at FDR 0.05 level.

**Figure 5:**
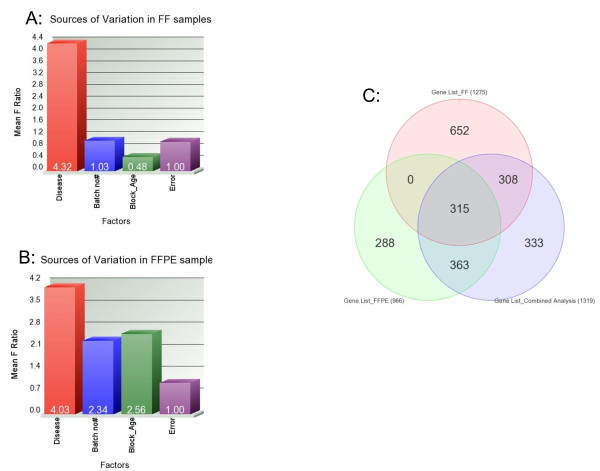
**Sources of variation & lists of differentially expressed genes in breast cancer**. **A. **Estimated measures of significance of the sources of variation in gene expression data in FF samples. **B. **Estimated measures of significance of the sources of variation in gene expression data in FFPE samples. **C. **Venn diagram showing overlap between three lists of differentially expressed genes (at FDR 0.05 level and at least 2-fold) in breast cancer tissue compared to corresponding adjacent normal breast tissue from three different analyses -using only FF samples (top center, showing 1275 genes), using only FFPE samples (bottom left, showing 966 genes) and using both the FF and FFPE samples in a combined analysis (bottom right, showing 1319 genes).

In the next step, we analyzed the data separately for FF and FFPE samples to evaluate the significance of "batch effect" and "sample block age" as sources of variation in the overall gene expression data. The F-ratio for these factors in these two types of sample storage methods are shown in Figure 5A and Figure 5B, respectively. The data shows that both the "batch effect" and "block age" contributed more significantly as the source of variation in overall expression data in the FFPE samples. However, by study design we assayed the paired samples on the same chip to minimize batch-effect. In paired analysis of the FF samples (tumor & adjacent normal), a total of 1275 genes were differentially expressed in breast tumor tissue by at least 2-fold at FDR 0.05 level (503 up- and 772 down-regulated in tumor). On the other hand, using the same criteria in FFPE samples, a total of 966 genes were differentially expressed in breast tumor (346 up-and 620 down-regulated in tumor).

Figure 5C shows the overlap of differentially expressed genes in the FF and FFPE samples and combined analysis. Only 315 genes were picked in both FF and FFPE samples, and all these 315 were also picked-up in the combined analysis. In other words, only one-third of the differentially expressed genes in tumor detected in FF samples could be picked up in FFPE samples and only one-fourth of the differentially expressed genes in tumor found in FFPE samples would be picked up in FF samples. Having this in mind, we further analyzed the lists of differentially expressed genes for GO-Enrichment to see if genes of similar GO- groups were found to be enriched in these lists derived from FF and FFPE samples.

### How do these gene lists compare to breast cancer or multiple cancer signatures from prior studies

Ein-dor et al [17] correctly described earlier that several microarray studies yielded gene sets whose expression profile successfully predicted survival, but the overlap between these gene sets was very low. We compared our three gene lists (FF, FFPE and from combined analysis) with some of the published data related to prognosis/survival [16,18-21] in breast and/or other cancer and two commercially available gene sets for real-time PCR assays from SA BioScience http://www.sabiosciences.com/ArrayList.php. It was not surprising to see different degree of overlap [see Table 2]. However it was interesting to see the predictive accuracy of those different gene lists from different studies or sources in our data set. For example, although only 4 out of 51 genes in BCGD list [16] was common with our 1319 genes, expression pattern of those 51 genes could correctly identify 39 out of 51 samples included in our study (predictive accuracy 76.53%). On the other hand, 10 out of 21 genes of the Martin KJ et al [18] list (almost 50%) overlapped with our list from combined analysis; however prediction based on expression of those 21 genes in our data set was just slightly higher at 80.23%.

**Table 2:** Overlap between our gene lists and the lists from other studies &/or sources

		**Martin et al **[18]	Multiple cancer gene list **	SA Bioscience Cancer Panel	SA Bioscience Breast Cancer panel	**Baasiri et al. (BCGD) **[16]
		**n = 21**	**n = 184**	**n = 83**	**n = 83**	**n = 51**

						

List from combined analysis, n = 1319	overlap	10	18	8	10	4
	
	% correct*	80.23	88.07	80.30	82.15	76.38

						

List from analysiss of FF samples, n = 1275	overlap	2	13	5	2	5
	
	% correct*	75.64	79.80	76.92	75.64	83.65

						

List from analysiss of FFPE samples, n = 966	overlap	7	10	8	9	1
	
	% correct*	84.61	88.46	88.46	80.76	76.92

*All the genes in the external list were used in a model to predict the tissue type (tumor or normal) in our data set to calculate the % correct rate. For all models, nearest centroid with equal prior probability was used as classification method. ** Gene lists are combined from Su et al [20], Yu et al [21] and Ramaswami et al.[19]

### GO Enrichment Analyses of the lists of differentially expressed genes in breast cancer tissue compared to normal breast tissue in FF and FFPE samples

**Figure 6:**
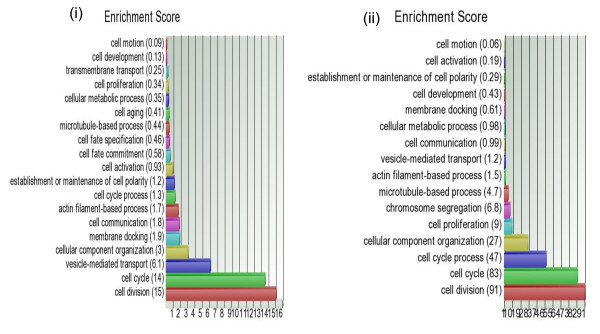
**GO-Enrichment for up-regulated genes**. GO-Enrichment analysis of the up-regulated genes for "cellular process" under the broad category "Biological process" in FF samples (on left) and in FFPE samples (on right). Different GO-terms are shown in vertical axis with Enrichment score in parenthesis.

**Figure 7:**
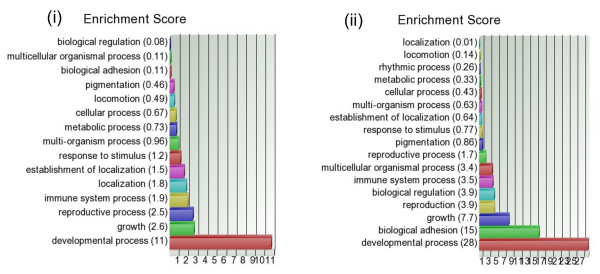
**GO-Enrichment for down-regulated genes**. GO-Enrichment analysis of the down-regulated genes for "cellular process" under the broad category "Biological process" in FF samples (on left) and in FFPE samples (on right).

The GO-Enrichment analysis tests if the genes found to be differentially expressed (at individual gene level) in a given list fall into a Gene Ontology category more often than expected by chance. Enrichment score was calculated as the negative log of the p-value of the test statistics. Hence, higher enrichment scores represent lead functional GO-groups. The functional groupings are sometimes easier to understand than pathways. The scores are used to rank the functional groupings. In GO, the genes are categorized into different GO-terms in three ways: (a) by "biological process", (b) by "molecular function" and (c) by "cellular components". First, we used GO-Enrichment analysis for the two lists of up-regulated genes in breast tumor derived from analysis of FF and FFPE samples. Figure 6(i) and Figure 6(ii) show hierarchical clustering of the enrichment scores for the "cellular process" category under the broad term "Biological process" for FF and FFPE samples respectively. It may be noted that in both the lists derived from FF and FFPE samples, the GO-term "cell division" and "cell cycle" were the top-ranking lead groups. This is very much biologically relevant with respect to any carcinogenesis in general. In fact, enrichment scores of "cell division" and "cell cycle" related genes constituted 56.87% of the total score for the "cellular process" for the up-regulated genes found in FF samples and 63.2% of the up-regulated genes found in FFPE sample. Similarly, we looked at the GO-Enrichment analysis for the down-regulated gene lists in tumor derived from FF and FFPE samples (see Figure 7(i) and Figure 7(ii) respectively for "biological process" categorization). The top-ranking functional lead groups show similarity between the gene lists derived from FF and FFPE samples. Similarly, under the broad category "Cellular components", the GO-term "extracellular region" comes up in both FF as well as FFPE samples (results not shown), emphasizing the significance of alteration in the extra-cellular matrix in breast carcinogenesis.

Therefore our findings suggest that the analysis of FFPE samples does not identify the exact same genes that would have been identified by analyzing FF samples, but at least, the list shows some similarity in terms of enrichment of GO-terms representing the lead functional groups of genes. In other words, FFPE samples may not be ideal for picking individual target gene(s), but may be used to identify the lead functional group(s) of genes that are differentially expressed in tumor.

For the up-regulated genes in breast tumor, that we found by analyzing the FF samples (and also in FFPE samples), if we looked at the enrichment of GO-terms under all the three major groups together - "biological process", "molecular function" and "cellular components", the top most ranking GO-term was "**nucleosome**" under "cellular components". All the up-regulated genes under this GO-term are from the same family of genes that are related to HIST1 and HIST2 proteins.

For the down-regulated genes in breast tumor, that we found by analyzing the FF samples, if we look at the enrichment of GO-terms under all the three major groups - "biological process", "molecular function" and "cellular components", the top ranking GO-term was "**regulation of epithelial cell proliferation**" under the "biological process". This finding is also very much relevant in the context of breast cancer pathogenesis. These down-regulated genes include *FGF2, FGF9, APC, IGF1, CDKN2B, NOTCH1, LAMC1, LAMB1, NKX3-1, TBX18, and GAS1*.

**Figure 8:**
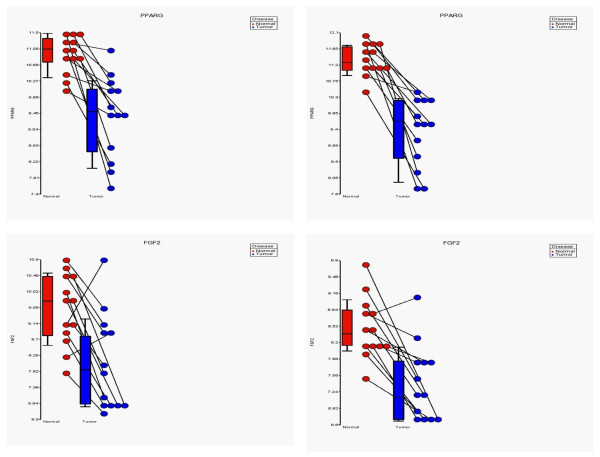
**Dotplot for *PPARG *and *FGF2 *in FF and FFPE samples**. Dot plots for "*PPARG*" are shown in upper panel and that of "*FGF2*" in the lower panel. In each panel data from FF samples are shown on the left plot and that of FFPE is shown on the right plots. In each dot plot, corresponding samples are joined by straight lines (indicating same patient), the gene expression in normal tissue is shown in red on left side and that of tumor tissue is shown in blue on right side of each dot plot. Each box extends from 25^th ^to 75^th ^centile with a central mark for 50^th ^centile; the whiskers show 10^th ^and 90^th ^centile for each set of data.

We performed GO-Enrichment analysis of the 315 genes common in all the three ANOVA analyses - (a) FF samples, (b) FFPE samples and in (c) combined analysis. Subsequent hierarchical clustering suggested that a number of those genes (including *PPARG, FGF2, APOB, CRHBP, CETP, and RXRG*) were significantly associated with breast cancer that appeared repeatedly in different GO-terms. For example, *PPARG *was significant in the GO-terms "anatomical structure development", "organ development", "developmental maturation", "lipid metabolism", "regulation of biological quality", "transcription factor activity", "molecular transducer activity", "drug binding", and "lipid binding". The dot plot in Figure 8 shows the expression of *PPARG *and *FGF2 *in paired samples (breast tumor and adjacent normal tissue; corresponding samples from same patient are connected by line) preserved as FF as well as FFPE sections. *PPARG *is shown to be down-regulated in breast cancer in all the patients and *FGF2 *is shown to be down-regulated in breast cancer in all but one patient in both FF and FFPE sections.

### Interaction between "tissue type" and "sample storage"

**Figure 9:**
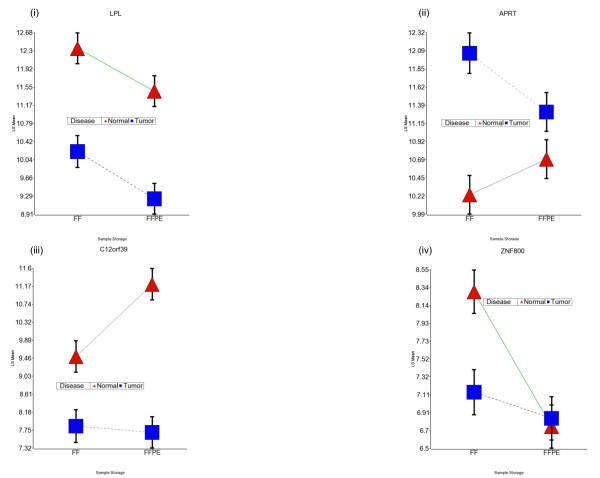
**Examples of interaction between "tissue type" and "sample storage"**. Error bar plots for least square means for differential gene expression data for (i) *LPL *(ii) *APRT*, (iii) *C12 orf 139 and *(iv) *ZNF 800 *genes. LPL shows the example with no interaction between "tissue type" and "sample storage", where as the other three genes show the examples of significant interaction - i.e., the differential expression in tumor tissue is significantly affected by the storage type. In each plot, breast tumor tissue is shown as blue square and adjacent normal breast tissue as red triangle; data from FF samples are presented on the left side and that of FFPE samples on the right side. The error bars represent standard error of mean.

In mixed model ANOVA, we also entered the interaction term "tissue type (tumor/normal) × sample storage (FFPE/FF)" to identify the genes that show a different degree, or even direction, in differential expression in tumor compared to normal tissue for FF or FFPE samples. There were a total of 772 genes that showed significant interaction at FDR 0.05; of which 178 also had differential expression in tumor at FDR 0.01 with at least 1.4-fold change (see Table 3). The present study suggests that differential expression of these genes in tumor should be cautiously interpreted if FFPE samples are studied. Figure 9(i) shows differential expression of a gene (*LPL*) without any significant interaction. Expression of the *LPL *gene was down-regulated in tumor tissue compared to adjacent normal tissue in an identical fashion in both FF as well as FFPE samples. On the other hand, Figure 9(ii-iv) shows the examples of the genes which show significant interaction, i.e. degree of differential expression in tumor (compared to normal), is significantly influenced by the storage. For example, if we look at the expression of *ZNF800 *gene [Figure 9(iv)] in FF tissue, we would notice significant down-regulation in tumor, but if we look at FFPE samples, there was no differential expression in tumor.

**Table 3:** List of 178 genes that show significant "Tissue * Storage" interaction and are also differentially expressed in breast cancer tissue at FDR 0.05 level with at least 1.4 fold change in the combined analysis.

*GENE_SYMBOL*	*p-value (Disease)*	*p-value (Storage)*	*p-value (Case ID)*	p-value (Disease * Storage)	*Fold- Change (Tumor vs. Normal)*	*Fold-Change (FFPE vs. FF)*
*WRB*	1.78E-03	3.40E-09	7.35E-07	6.59E-06	-1.41	-2.21

*TPCN1*	1.10E-05	8.55E-01	4.37E-05	6.85E-06	1.66	1.02

*ANKFY1*	1.95E-06	7.64E-01	1.04E-04	7.24E-06	1.42	1.02

*CYP2U1*	2.62E-08	8.80E-10	1.16E-03	1.01E-05	-1.98	-2.22

*SMTN*	9.55E-04	1.47E-01	2.11E-04	1.24E-05	1.47	1.17

*LNX1*	2.10E-05	7.85E-04	3.50E-02	1.30E-05	-1.90	-1.62

*CBL*	2.48E-05	8.60E-06	2.30E-05	1.71E-05	-1.62	-1.68

*KLHL2*	9.67E-04	1.51E-10	2.73E-07	1.72E-05	-1.46	-2.57

*AKAP8L*	1.75E-07	1.64E-01	6.17E-04	1.87E-05	1.75	1.13

*RFXANK*	1.13E-07	3.60E-01	9.57E-05	2.32E-05	1.65	1.07

*CTBP1*	1.41E-06	6.11E-05	7.37E-06	2.94E-05	1.54	1.40

*HELB*	7.51E-08	6.99E-06	3.10E-03	3.78E-05	2.89	2.28

*CCDC101*	5.10E-05	7.93E-01	1.24E-02	4.39E-05	1.51	-1.02

*SFRS16*	6.63E-04	7.91E-05	2.77E-02	4.66E-05	1.58	-1.73

*C2orf33*	1.98E-03	5.92E-02	5.27E-11	4.77E-05	-1.41	-1.22

*SMARCB1*	7.18E-10	1.10E-02	2.94E-04	5.15E-05	1.81	1.21

*TOLLIP*	4.00E-07	9.39E-02	9.85E-05	5.16E-05	2.58	1.30

*PLEC1*	4.76E-06	1.18E-09	3.41E-05	6.28E-05	1.71	2.26

*EBNA1BP2*	2.76E-05	5.97E-03	4.00E-05	6.47E-05	1.41	-1.23

*EFTUD2*	4.09E-06	1.28E-03	6.33E-02	6.59E-05	1.40	1.24

*POLDIP3*	1.58E-07	2.39E-01	6.11E-03	8.18E-05	1.44	1.07

*RPL6*	1.21E-10	3.21E-04	6.06E-04	8.22E-05	2.53	1.51

*ITIH5*	4.60E-05	6.68E-01	1.87E-02	8.39E-05	-1.56	1.04

*GBF1*	1.18E-08	4.35E-03	1.05E-03	1.00E-04	1.97	1.32

*MAEA*	2.50E-08	4.61E-02	7.21E-02	1.03E-04	1.68	1.16

*CCT2*	1.17E-08	6.92E-05	2.15E-04	1.15E-04	1.71	1.39

*SMC6*	5.61E-04	4.28E-03	1.65E-05	1.31E-04	-1.51	-1.40

*C19orf61*	8.87E-08	4.16E-01	2.55E-02	1.31E-04	1.99	1.09

*VPS28*	1.63E-06	1.44E-01	7.92E-04	1.34E-04	1.62	-1.13

*QTRT1*	2.39E-04	4.20E-01	8.43E-01	1.37E-04	1.60	1.10

*RPL12*	5.18E-06	1.54E-01	4.17E-02	1.40E-04	1.49	-1.11

*CNOT3*	4.87E-07	9.43E-01	1.48E-03	1.43E-04	1.72	-1.01

*BANF1*	7.48E-09	1.49E-03	1.84E-04	1.51E-04	1.57	1.23

*AATF*	1.92E-07	8.25E-02	1.08E-03	1.58E-04	1.76	-1.17

*NDUFAF1*	2.59E-05	8.26E-11	1.35E-01	1.60E-04	-1.49	-2.12

*SDCCAG1*	2.36E-05	1.39E-01	3.91E-04	1.69E-04	1.50	-1.13

*BNIP1*	2.36E-05	5.09E-04	5.73E-03	1.72E-04	1.53	1.39

*LOC728758*	3.19E-04	1.24E-02	6.97E-03	1.74E-04	-1.82	1.49

*RPL23AP7*	1.52E-06	5.66E-10	1.00E-01	1.79E-04	1.41	-1.65

*LSM11*	7.45E-07	5.36E-01	1.06E-02	1.87E-04	1.58	1.05

*CLPTM1*	3.20E-06	6.03E-04	1.21E-04	1.88E-04	1.58	1.37

*POGZ*	8.70E-05	5.28E-07	2.38E-02	1.91E-04	1.48	1.71

*SC5DL*	7.77E-07	3.64E-02	2.47E-08	2.02E-04	-1.61	1.19

*XRCC6*	9.50E-07	6.25E-01	4.42E-03	2.13E-04	1.83	1.05

*LOC205251*	7.83E-04	2.26E-04	5.18E-03	2.41E-04	1.57	-1.66

*PLEKHG1*	1.89E-06	5.93E-05	2.48E-04	2.42E-04	-2.18	-1.87

*SPATA22*	1.33E-05	1.92E-02	4.97E-04	2.48E-04	-2.62	-1.60

*CENTB5*	5.14E-05	7.22E-02	5.84E-04	2.54E-04	1.67	1.23

*ZCCHC8*	8.69E-06	1.74E-02	1.17E-02	2.67E-04	1.47	1.20

*KLHL20*	4.97E-06	9.02E-02	5.65E-01	2.77E-04	-1.62	1.17

*BANK1*	2.22E-08	3.82E-05	6.06E-03	2.93E-04	-2.95	-2.03

*SGTA*	3.52E-04	5.78E-02	3.09E-04	2.93E-04	1.57	-1.25

*ACIN1*	1.88E-05	4.24E-10	4.33E-03	2.98E-04	1.45	1.91

*RCE1*	2.27E-08	3.07E-05	4.81E-03	3.16E-04	1.81	1.49

*NUDT13*	1.37E-05	2.79E-06	4.24E-04	3.33E-04	-1.45	-1.51

*GPR64*	4.44E-09	1.13E-04	1.69E-03	3.33E-04	-2.75	-1.77

*STAMBPL1*	4.63E-04	1.47E-05	1.28E-01	3.44E-04	-1.41	-1.56

*SHKBP1*	2.42E-06	1.23E-07	8.23E-04	3.71E-04	1.49	1.60

*C9orf86*	2.04E-05	6.48E-01	1.82E-03	3.81E-04	2.40	-1.09

*ECHDC1*	2.80E-06	3.82E-03	2.11E-06	3.88E-04	-2.04	-1.49

*EDF1*	5.10E-06	2.30E-01	8.34E-05	4.12E-04	1.42	1.08

*PRMT7*	2.10E-04	8.12E-01	6.42E-03	4.12E-04	2.11	1.04

*HERC2P2*	7.56E-04	9.75E-03	6.71E-01	4.36E-04	1.85	1.58

*TOPORS*	1.21E-04	2.75E-06	1.66E-06	4.37E-04	-1.64	-1.90

*RCBTB2*	1.80E-04	2.21E-06	1.76E-05	4.38E-04	-1.53	-1.77

*BRF1*	4.92E-06	5.96E-02	5.32E-05	4.40E-04	1.92	1.26

*LOC440836*	2.48E-04	3.96E-09	2.34E-04	4.45E-04	2.13	4.21

*ZNF800*	2.21E-03	4.28E-06	1.26E-05	4.48E-04	-1.46	-1.87

*IGBP1*	3.76E-04	1.23E-04	7.30E-02	4.62E-04	1.77	1.87

*SLCO1C1*	7.65E-11	1.40E-05	7.92E-06	4.71E-04	-2.18	-1.53

*CCDC97*	2.50E-05	2.67E-04	2.14E-02	4.73E-04	1.65	-1.52

*ARMCX1*	2.36E-05	1.27E-05	6.04E-06	5.06E-04	-1.87	-1.92

*PCAF*	6.56E-06	2.64E-09	7.50E-05	5.09E-04	-1.53	-1.89

*INPP5A*	1.98E-03	1.20E-03	4.68E-04	5.26E-04	-1.45	-1.48

*FGFR1OP2*	1.03E-04	2.73E-03	5.53E-05	5.32E-04	-1.47	-1.33

*ZBTB37*	1.07E-03	8.73E-01	3.47E-05	5.33E-04	1.61	-1.02

*ERGIC1*	5.17E-07	1.05E-02	1.77E-02	5.42E-04	1.64	-1.24

*THAP10*	3.18E-04	1.96E-03	2.71E-02	5.62E-04	-1.70	-1.56

*CSTF2T*	1.64E-03	2.02E-10	3.38E-05	5.71E-04	-1.55	-3.08

*CTNND1*	1.96E-06	5.30E-05	2.25E-01	5.72E-04	1.49	1.38

*CKS2*	2.43E-05	9.21E-04	2.19E-01	5.73E-04	2.73	-2.11

*C7orf26*	1.34E-04	4.08E-01	5.39E-02	5.75E-04	1.55	1.09

*PRKAR1B*	6.89E-04	1.47E-01	1.68E-01	5.79E-04	1.76	1.25

*NF2*	8.23E-08	1.54E-01	8.02E-03	5.88E-04	1.60	1.11

*UBR4*	2.48E-05	9.60E-08	5.01E-02	6.22E-04	1.53	1.80

*UFM1*	2.30E-05	5.45E-01	4.26E-09	6.36E-04	-1.45	-1.05

*ARID1B*	1.38E-03	1.01E-03	5.96E-01	6.39E-04	1.43	1.44

*TSPAN12*	7.09E-06	1.31E-07	2.74E-06	6.41E-04	-2.17	-2.63

*TXNDC5*	4.99E-08	2.44E-08	6.66E-04	6.45E-04	1.77	1.81

*CLTC*	9.24E-05	7.98E-11	3.76E-04	6.57E-04	-1.45	-2.16

*C17orf38*	1.38E-03	1.16E-01	3.79E-01	6.58E-04	1.84	1.33

*SHOC2*	3.10E-04	3.40E-08	9.38E-03	6.73E-04	-1.46	-1.94

*MAN2B1*	1.59E-04	1.04E-04	1.87E-02	6.88E-04	1.47	1.49

*FUCA2*	3.16E-04	1.16E-03	1.02E-02	6.92E-04	-1.74	-1.63

*MED24*	4.68E-07	7.29E-01	1.60E-03	7.36E-04	1.82	-1.03

*C1orf86*	2.05E-08	8.20E-02	2.70E-05	7.73E-04	1.85	-1.16

*TUBGCP6*	2.57E-05	2.13E-02	1.35E-02	7.77E-04	1.47	1.21

*PLCB3*	3.60E-05	1.98E-02	3.19E-02	7.84E-04	1.50	-1.23

*PAN3*	1.27E-05	3.57E-09	1.41E-04	7.84E-04	-1.44	-1.75

*BDH2*	8.19E-06	1.98E-06	6.92E-03	7.87E-04	-2.50	-2.71

*NR3C2*	6.25E-07	2.97E-11	1.65E-03	7.93E-04	-2.29	-3.68

*TRIM44*	1.74E-04	2.28E-03	9.50E-03	8.03E-04	-1.80	-1.59

*RBM42*	3.45E-08	8.38E-01	1.16E-04	8.14E-04	1.40	-1.01

*IQCB1*	1.52E-06	7.01E-04	5.37E-03	8.21E-04	1.61	1.36

*TMEM106B*	4.98E-04	1.77E-05	2.12E-14	8.24E-04	-1.51	-1.70

*C9orf142*	5.85E-12	4.64E-03	4.62E-05	8.46E-04	1.90	-1.21

*ADRM1*	2.00E-05	9.59E-01	1.31E-04	8.49E-04	1.40	1.00

*NOC4L*	6.75E-04	3.10E-04	2.61E-03	8.54E-04	1.46	1.50

*ZFYVE16*	6.13E-04	1.66E-02	1.16E-03	8.63E-04	-1.54	-1.33

*HPS1*	1.80E-04	5.28E-04	1.53E-03	8.68E-04	1.72	1.64

*RAB4B*	2.82E-05	6.19E-06	1.50E-04	8.90E-04	2.82	3.14

*MUT*	2.14E-05	2.01E-06	7.54E-05	8.96E-04	-1.66	1.79

*HNRNPL*	4.63E-06	5.51E-03	1.18E-01	9.18E-04	1.61	1.30

*LIN7C*	2.12E-04	1.28E-06	5.96E-07	9.22E-04	-1.61	-1.96

*VCPIP1*	1.49E-03	3.15E-06	5.03E-02	9.42E-04	-1.42	-1.76

*DLD*	9.70E-04	8.80E-08	1.75E-03	9.49E-04	-1.49	-2.09

*DLAT*	2.09E-05	3.75E-02	1.21E-06	9.73E-04	-1.54	-1.21

*FGF13*	2.54E-04	6.38E-08	8.76E-03	9.89E-04	-1.75	-2.55

*C5orf41*	7.05E-05	1.75E-09	5.73E-03	1.05E-03	-1.48	-2.01

*TMEM134*	2.70E-08	5.91E-02	1.18E-03	1.05E-03	2.00	-1.21

*C12orf39*	4.41E-12	3.12E-03	2.44E-05	1.06E-03	-6.13	1.75

*YAP1*	2.16E-06	5.80E-07	1.46E-04	1.08E-03	-1.58	-1.64

*NOC2L*	2.15E-04	2.66E-02	6.49E-04	1.10E-03	1.63	-1.32

*MAL2*	3.09E-04	8.75E-06	2.33E-02	1.12E-03	1.87	-2.25

*PTPLAD1*	1.17E-05	8.28E-10	2.11E-02	1.13E-03	1.43	-1.78

*SDC3*	1.95E-03	1.05E-01	9.52E-02	1.14E-03	1.48	1.21

*CCNL2*	2.72E-04	6.33E-01	1.73E-02	1.15E-03	1.60	1.06

*ARHGEF15*	8.73E-06	8.29E-01	4.72E-02	1.18E-03	-2.02	1.03

*IFIT5*	7.61E-04	7.57E-12	1.27E-02	1.18E-03	-1.45	-2.76

*PDGFC*	1.44E-05	1.94E-08	1.92E-06	1.19E-03	-1.74	-2.22

*HIP1R*	5.96E-04	7.12E-06	2.55E-02	1.19E-03	1.50	1.76

*FBXL18*	4.98E-04	1.16E-02	9.70E-02	1.20E-03	1.68	1.44

*PDLIM7*	4.33E-05	1.22E-04	1.34E-02	1.22E-03	1.70	1.63

*LOC90379*	2.62E-04	2.22E-04	1.45E-03	1.23E-03	1.73	1.74

*CLDN5*	2.33E-03	1.21E-03	4.47E-01	1.24E-03	-1.90	-2.00

*ZNF558*	6.51E-04	3.18E-06	2.49E-06	1.25E-03	-1.41	-1.67

*ATP8A1*	9.49E-04	3.31E-03	1.32E-03	1.29E-03	1.86	1.72

*HTF9C*	8.06E-05	7.73E-03	3.66E-03	1.30E-03	1.62	1.36

*TOP1MT*	1.04E-05	7.89E-04	1.88E-04	1.30E-03	1.59	1.39

*DDIT3*	3.71E-05	3.25E-01	1.58E-02	1.31E-03	1.64	1.11

*HSF1*	7.00E-05	4.86E-03	1.29E-02	1.32E-03	2.53	1.86

*APRT*	5.71E-08	3.55E-01	1.22E-04	1.33E-03	2.36	-1.12

*C17orf49*	1.48E-06	2.40E-03	8.03E-04	1.35E-03	1.45	-1.23

*GABARAPL2*	7.79E-05	1.43E-08	3.81E-05	1.36E-03	-1.53	-2.00

*PRKAA1*	6.97E-07	5.50E-01	4.02E-03	1.37E-03	-1.53	-1.04

*PLXNA4B*	2.90E-06	1.93E-05	1.03E-02	1.40E-03	-3.29	2.87

*SPPL2B*	1.84E-04	9.70E-01	8.27E-03	1.46E-03	1.65	-1.00

*IL17RC*	4.01E-04	2.18E-01	2.42E-04	1.46E-03	1.73	1.19

*C9orf66*	5.23E-04	3.12E-03	1.62E-01	1.47E-03	-1.56	-1.45

*PPAT*	3.48E-05	4.79E-01	5.33E-02	1.47E-03	1.55	1.07

*ENO3*	2.55E-06	7.64E-03	5.28E-04	1.47E-03	1.94	-1.40

*NUP85*	1.61E-07	5.16E-03	1.79E-01	1.48E-03	1.65	-1.26

*JMJD1B*	5.67E-05	2.06E-08	2.51E-06	1.49E-03	-1.64	-2.19

*C20orf149*	4.13E-08	4.50E-03	3.19E-02	1.49E-03	1.89	1.32

*LRRTM2*	7.98E-09	4.21E-04	1.94E-06	1.50E-03	-2.43	-1.58

*NSUN5*	1.64E-06	2.55E-02	6.91E-03	1.52E-03	1.44	1.16

*C15orf48*	1.08E-04	3.77E-07	2.00E-04	1.52E-03	1.77	-2.25

*SHROOM2*	5.44E-05	2.24E-02	6.12E-01	1.52E-03	2.38	-1.57

*TMCC1*	8.49E-04	1.92E-01	1.62E-03	1.53E-03	1.42	1.14

*TMEM16C*	1.82E-05	4.87E-05	5.35E-02	1.54E-03	-1.99	-1.90

*MRPL53*	3.95E-04	1.70E-01	2.16E-03	1.54E-03	1.42	-1.13

*IGSF3*	1.45E-03	3.95E-08	7.32E-03	1.61E-03	1.73	-3.02

*ALG1*	1.09E-07	4.88E-04	3.86E-02	1.61E-03	2.42	1.67

*ICOSLG*	8.82E-04	6.33E-04	1.25E-02	1.72E-03	1.57	1.59

*PHC2*	1.51E-04	1.36E-01	6.48E-02	1.72E-03	1.66	1.20

*UBFD1*	2.24E-08	3.23E-01	8.08E-02	1.78E-03	1.99	1.10

*RPS3*	5.32E-06	1.85E-02	8.89E-03	1.88E-03	1.53	1.22

*CTF8*	1.51E-05	1.01E-08	1.58E-02	1.90E-03	1.83	2.44

*TCOF1*	1.06E-03	1.69E-04	6.12E-05	1.90E-03	1.47	1.58

*LOC407835*	1.67E-05	4.40E-03	1.22E-03	1.91E-03	1.80	1.43

*MIB2*	1.02E-03	7.29E-03	1.51E-02	1.91E-03	1.45	1.34

*SPATA13*	2.19E-03	1.67E-08	4.23E-01	1.95E-03	1.44	2.22

*C11orf73*	6.88E-04	2.78E-15	1.69E-02	1.97E-03	-1.51	-4.38

*PPAN*	6.63E-04	5.04E-01	3.06E-03	1.98E-03	1.56	1.08

*LACTB2*	1.98E-06	4.55E-01	5.63E-06	1.98E-03	-2.06	1.10

*FVT1*	5.84E-06	5.82E-04	2.24E-07	1.99E-03	-1.77	-1.50

*NRARP*	1.04E-03	1.58E-04	7.55E-05	2.01E-03	2.14	2.47

*C10orf97*	1.31E-07	1.14E-01	3.59E-08	2.09E-03	-1.82	1.16

### Expression of known "clinically relevant" genes in our data

From a therapeutic point of view, determination of *HER2*, estrogen receptor (*ESR*) and progesterone receptor (*PGR*) gene expressions is very helpful in decision-making for therapeutic regimens [22]. We therefore looked at these genes in particular in our data set (data not shown). Expression of the *ESR1 *gene (*ESR1*) was significantly affected by FFPE storage. Expression of the *ESR2 *and *PGR *genes were very minimally affected by storage, and that of *HER2 *was not affected at all. We found *HER2 *to be up-regulated by 1.5 fold (p = 0.0054) and *PGR *to be down-regulated by 1.9 fold (p = 0.006) in tumor compared to adjacent normal tissue.

### Do we see same "gene sets" to be differentially expressed in breast tumor tissue compared to corresponding adjacent normal breast tissue in FF and FFPE samples?

After looking into the differential expression at individual gene level, we also looked for differential expression of different "gene sets" in breast cancer tissue by using the Gene Set Enrichment Analysis (GSEA) as well as GO-ANOVA. GSEA of the FF samples showed that a total of 100 "gene sets" (each representing different GO description terms) were differentially expressed (p-value = < 0.05) in breast cancer tissue compared to adjacent healthy tissue. On the other hand, GSEA of the FFPE samples showed a total of 440 "gene sets" were differentially expressed at p-value = < 0.05 in breast cancer FFPE tissue compared to adjacent healthy FFPE tissue. Only 38 "gene sets" (i.e. 38% "gene sets" from of the FF analysis and only 8.64% from FFPE analysis) were common in both the analyses. Therefore, GSEA also suggested the need for caution for interpretation of gene expression data from FFPE samples.

GO-ANOVA of the FF samples showed that a total of 883 "gene sets" (each representing different GO description terms) were differentially expressed at FDR 0.05 in breast cancer tissue compared to adjacent healthy tissue. On the other hand, GO-ANOVA of the FFPE samples showed that 1,034 "gene sets" were differentially expressed at FDR = < 0.05 in breast cancer FFPE tissue compared to adjacent healthy FFPE tissue. A total of 641 "gene sets" (i.e. 72.59% gene sets from FF and 61.99% from FFPE analysis) were common in both the analyses. This result also suggested the need for caution for interpretation of gene expression data from FFPE samples.

**Figure 10:**
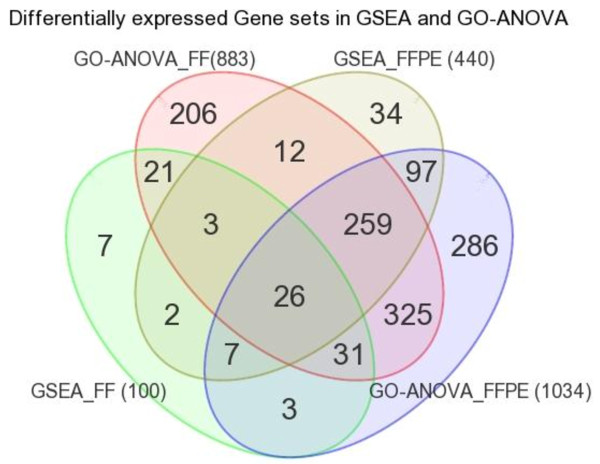
**Overlap between GSEA and GO-ANOVA**. Venn diagram showing the overlap between four lists of differentially expressed "gene sets" in breast cancer derived from GSEA and GO-ANOVA in FF and FFPE samples.

The overlaps of these results from GSEA and GO-ANOVA in FF and FFPE samples are presented in Figure 10. In general, GO-ANOVA could detect larger number of differentially expressed gene sets (GO-description terms), however it may be noted that most of the gene sets detected by GSEA were also detected by GO-ANOVA - 81.0% in FF and 88.4% in FFPE samples. There was a total of 26 "gene sets" that was common to all four analyses. Those 26 gene sets are shown in Table 4. It may be noted that genes related to "transforming growth factor beta receptor signaling pathway" were down-regulated (positive NES in this case) and genes related to "nucleosome" were up-regulated (negative NES in this case) in breast cancer. It may be noted that GO-Enrichment analysis of the differentially expressed gene lists also showed highest enrichment score for genes related to "nucleosome".

**Table 4:** Results from GSEA for breast cancer in FF samples. The following 26 gene sets (GO-terms) were common in all the four lists shown in figure-4(v)

Gene Set Description	# of Genes	ES*	NES**	p-value
metalloexopeptidase activity	37	0.5273	1.6927	0.0000

transforming growth factor beta receptor signaling pathway	67	0.4600	1.6388	0.0192

fatty acid metabolic process	116	0.3829	1.5961	0.0000

sterol transport	26	0.4747	1.5879	0.0417

cholesterol transport	25	0.4770	1.5853	0.0208

vitamin metabolic process	22	0.5190	1.5649	0.0204

regulation of insulin secretion	26	0.4652	1.5559	0.0000

regulation of biomineral formation	26	0.4641	1.5288	0.0000

muscle cell differentiation	40	0.4888	1.5192	0.0408

vitamin binding	121	0.3420	1.4857	0.0385

regulation of blood vessel size	43	0.4805	1.4810	0.0408

regulation of tube size	43	0.4805	1.4810	0.0408

BMP signaling pathway	36	0.5195	1.4783	0.0426

regulation of cell proliferation	171	0.3540	1.4709	0.0200

glucose metabolic process	42	0.3829	1.4667	0.0217

blood circulation	46	0.4345	1.4544	0.0204

branching morphogenesis of a tube	55	0.4291	1.4160	0.0208

regulation of hormone secretion	36	0.3898	1.4101	0.0385

magnesium ion binding	418	0.2887	1.3951	0.0000

GTPase binding	85	0.3092	1.3766	0.0000

response to drug	81	0.3105	1.3461	0.0364

carbohydrate binding	301	0.2844	1.3073	0.0204

small GTPase binding	80	0.2944	1.2867	0.0364

protein dimerization activity	433	0.2449	1.2408	0.0196

negative regulation of DNA metabolic process	23	-0.4876	-1.5264	0.0217

nucleosome	87	-0.6222	-1.5847	0.0417

*ES: Enrichment score, **NES: normalized enrichment score

## Discussion

Gene expression profiling of human cancer has proved valuable in cancer research leading not only to the identification of targets but also contributing to our understanding of the mechanisms of the process [23,24]. The application of microarrays is limited by the availability of fresh frozen tissue or the tissue preserved in RNAlater reagent. As FFPE samples are available in almost all the pathological laboratories and are often available in conjunction with clinical and follow-up data, they would be considered as the most valuable sources for microarray analysis [25], provided similar information can be obtained as would be expected from analyzing the FF samples. Because of fragmentation [3,26,27] and some other chemical modifications of RNA in FFPE samples, currently gene expression studies are largely limited to immuno-histochemical (IHC) staining and RT-PCR, which allow only a few genes to be amplified at a time [3,28,29]. In this paper we mainly focus on the use of FFPE samples in genome-wide gene expression experiments. We have tried to analyze the data for differential expression from different angles - at individual gene level, at "gene set" level and also used different statistical methods. In follow-up paper we would focus more on gene selection and relevance to breast cancer biology.

Like other investigators [5,28,30,31], we also observed high reproducibility across technical replicates regardless of the sample type. However, the concordance between the paired FF and FFPE samples was weaker in our study, which is also consistent with other studies [23,32]. The tissue archival age, or the "FFPE block age" is another factor for consideration. Cronin et al. compared frozen section breast tissue with FFPE samples of "various block ages" by RT PCR for 92 genes and found a 90% signal loss in FFPE samples [26]. Our data on the genome-wide level also suggested the significance of FFPE "block age" on gene expression data. Srinivasan et al. [2], and Karsten et al [33] and Masuda et. al [1] have reviewed in detail the effect of fixative and tissue processing on the content and integrity of nucleic acids. There are four types of reactions of formaldehyde with nucleic acids: (1) Addition reaction or methylolation - the N-H groups of primarily adenine and thiamine are converted to N-CH2-OH groups (methylol groups). The poly(A) tail on RNA is thus heavily methylolated leading to poor reverse transcription. This methylolation is possibly reversible through hydrolysis. (2) Cross-linking - methylolated bases can react with N-H groups (on proteins and nucleic acids) to form -N-CH_2__N- cross-links, which are not easily hydrolyzed. (3) Formation of apyrimidinic/apurinic sites - the N-glycosidic bond between A, C, G, T or U and the sugar backbone is broken, leaving a blank space in the sequence. This is not base-specific, but is not reversible. (4) Fragmentation - Formaldehyde catalyzes the hydrolysis of phosphodiester bonds, fragmenting strands of nucleic acids, which is also not reversible. Therefore, it was not a surprise to find differential gene expression in FFPE samples compared to FF samples in the present study.

Bibikova et al. used a smaller panel of the DASL assay with 16 pairs of FF and FFPE samples from healthy and tumor breast tissue and healthy and colon cancer tissue [6]. They found that FFPE samples had 50% less gene expression compared to matched FF samples, which may be due to RNA degradation related to fixation and storage [6]. The present study is one of the first few adequately powered, whole-genome DASL assays interrogating more than 18,000 genes that has compared the results of paired tumor and normal tissue from FF and FFPE samples.

Our findings suggest that the analysis of FFPE samples does not identify the exact same genes that would have been identified by analyzing FF samples, but at least, the list shows some similarity in terms of enrichment of GO-terms representing the lead functional groups of genes. In other words, FFPE samples may not be ideal for picking individual target gene(s), but may be used to identify the lead functional group(s) of genes that are differentially expressed in tumor. Findings of the differentially expressed genes in breast cancer were biologically meaningful. On the one hand the "cell cycle" & "cell division" related genes were up-regulated and on the other hand, genes related to "regulation of epithelial cell proliferation" were down-regulated. Genes involved in metalloexopeptidase activity, transforming growth factor beta signaling pathway, BMP signaling pathway, were found to be down-regulated in breast cancer.

As mentioned in the results section, some of the genes (including *PPARG *and *FGF2*) were found repeatedly in the lists of different GO-terms. Peroxisome proliferator-activated receptor-γ (*PPARG*), is expressed in a large number of human cancers, including breast, colon, stomach, prostate, pancreas, bladder, placenta, lung, chondrosarcoma and in leukemia [34,35]. Recently Jiang et al. showed that PPARG expression in immunohistochemistry was positively correlated to estrogen receptor status, inversely associated with histological grade and tumor size, and in survival analysis patients with higher *PPARG *expression had significantly better prognosis [36]. In the same direction, the present study showed evidence of down-regulation of *PPARG *in breast cancer tissue. GWAS using germ line DNA showed a significant association of SNP in the *FGFR2 *gene with breast cancer [37]. In the same line, in the present study, we also found *FGF2 *to be down-regulated in breast cancer. Another biologically relevant gene that we found to be differentially expressed in breast cancer was corticotropin releasing hormone binding protein (*CRHBP*). Corticotropin-releasing hormone is a potent stimulator of synthesis and secretion of preopiomelanocortin-derived peptides. Although CRH concentrations in the human peripheral circulation are normally low, they increase throughout pregnancy and fall rapidly after parturition. Maternal plasma CRH probably originates from the placenta. Human plasma contains a CRH-binding protein that inactivates CRH and may prevent inappropriate pituitary-adrenal stimulation in pregnancy.

An apparent weakness of the present study is the lack of gene expression data from breast tissue preserved in RNA stabilization buffer, which would have served as the gold standard against which FFPE samples could have been compared. However, 72% of the genes that we found to be differentially expressed in FFPE breast tissue compared to corresponding FF in present study were also differentially expressed in FFPE skin tissue compared to the "gold standard"- RNAlater preserved corresponding skin tissue sample (unpublished data from our group). One of the strengths of the present study is that it was adequately powered to detect differential expression arising from tissue storage (FFPE vs. FF) or disease status (tumor vs. adjacent normal tissue).

## Conclusion

In agreement with other studies using the DASL platform, our present study also suggests the usefulness of DASL chemistry to study gene expression in fragmented RNA samples. DASL can efficiently handle the fragmentation issue of RNA in FFPE samples. However, formalin fixation used in FFPE induces significant gene expression change in a number of genes, and these changes may differ in degree or even in direction between tumor and normal tissue. Therefore, FFPE samples should not be directly compared with FF samples and considerable caution must be taken when interpreting gene expression data from FFPE samples. Despite these constraints, we found a number of biologically meaningful, differentially expressed genes related to *HIST1*, *HIST2 *proteins, and some other such as *PPARG, FGF2, APOB, CRHBP, CETP, and RXRG *in breast cancer tissue compared to corresponding adjacent normal breast tissue. The validity of these specific observations, however, needs to be confirmed in future larger studies.

## Authors' contributions

MGK designed the study, performed data analysis and wrote the manuscript, FJ carried out the DASL assay and drafted the manuscript, SR processed the tissue samples, ran the real-time PCR and helped in DASL assay, RMP performed the prequalification and DASL assay, MA managed the IRB approval and helped in procurement of the tissue samples, HA conceived & designed the study, helped in manuscript, supported & coordinated the study. All authors read and approved the final manuscript.
